# Current State of Surgical Lighting

**DOI:** 10.1055/s-0040-1710529

**Published:** 2020-06-19

**Authors:** Jahnavi Curlin, Charles K. Herman

**Affiliations:** 1 Department of Medicine, University of California-San Francisco, San Francisco, California; 2 Department of Surgery, Department of Surgery, Geisinger Commonwealth School of Medicine, Scranton, Pennsylvania; 3 Division of Plastic Surgery, Division of Plastic Surgery, Lehigh Valley Health Network, Lehigh Valley Hospital-Pocono, East Stroudsburg, Pennsylvania

**Keywords:** surgical lighting, disposable instruments, lighted retractors, skin burns, overhead lighting

## Abstract

Surgical performance in the operating room (OR) is supported by effective illumination, which mitigates the inherent environmental, operational, and visual challenges associated with surgery. Three critical components are essential to optimize operating light as illumination: (1) centering on the surgeon's immediate field, (2) illuminating a wide or narrow field with high-intensity light, and (3) penetrating into a cavity or under a flap. Furthermore, optimal surgical illumination reduces shadow, glare, and artifact in visualization of the surgical site. However, achieving these principles is more complex than at first glance, requiring a detailed examination of the variables that comprise surgical illumination. In brief, efficacious surgical illumination combines sufficient ambient light with the ability to apply focused light at specific operative stages and angles. But, brighter is not always merely better; rather, a nuanced approach, cognizant of the challenges inherent in the OR theater, can provide for a thoughtful exploration of how surgical illumination can be utilized to the best of its ability, ensuring a safe and smooth surgery for all.

## Current Lighting Methods in Operating Rooms

Surgical performance in the operating room (OR) is supported by effective illumination, which mitigates the inherent environmental, operational, and visual challenges associated with surgery. Okoro et al describe three critical components to optimize operating light as illumination: (1) centering on the surgeon's immediate field, (2) illuminating a wide or narrow field with high-intensity light, and (3) penetrating into a cavity or under a flap. 1 Furthermore, optimal surgical illumination reduces shadow, glare, and distortion in visualization of the surgical site. However, achieving these principles is more complex than at first glance, requiring a detailed examination of the variables that comprise surgical illumination. In brief, efficacious surgical illumination combines sufficient ambient light with the ability to apply focused light at specific operative stages and angles. But, brighter is not always merely better; rather, a nuanced approach, cognizant of the challenges inherent in the OR theater, can provide for a thoughtful exploration of how surgical illumination can be utilized to the best of its ability, ensuring a safe and smooth surgery for all.

There are currently four predominant methods of illumination utilized in the surgical field: surgical lighting systems (SLS), lighted retractors, headlights, and operating microscopes. Current methods of illumination address the fundamental needs of illumination, largely intensity and control, in slightly different ways. For traditional open surgeries across surgical disciplines, SLS, commonly referred to simply as OR lights, are broadly utilized to illuminate the OR during procedures. Lighted surgical retractors, on the other hand, are relatively more recent innovations that provide in-field focused illumination targeted to the surgical site. To promote increased mobility and manipulation of the light field, however, surgeons may elect to wear headlights. Operating microscopes are exclusively utilized in microsurgery and provide the advantages of magnification and reverse illumination. Each illumination method carries its own distinct advantages and disadvantages, and use is dependent on the surgeon as well as the operation itself.

Current illumination methods are limited by the lack of mobility, repetitive and time-lengthy adjustments, sterilization, and contamination concerns, nonoptimal illumination, inefficiencies, and time delays. Knulst et al highlighted the ergonomic concerns around overhead lighting systems, noting that every 7.5 minutes the adjustment of a two-arm pendant luminaire system occurred. 2 The cited reason for initiating luminaire actions was to reestablish lighting at surgical sites, and at adjusted angles, particularly in large, narrow but deep, and multiple wounds. Knulst et al also emphasized that complications, such as mechanical issues, that were encountered during luminaire actions increased the median time of adjustment, thus adding to the overall duration of surgery.

In addition to the exact need for visualization, there is also a requirement for a nuanced approach to delivering light at the surgical site. Traditional OR lights often provide high-intensity, directional light, which serves as beneficial up to a threshold. A great amount of light directed toward a surgical site allows for increased reflection off structures, which provides for effective contrast. Contrast here refers to the ability to confidently differentiate between different structures at the surgical site, including microstructures. However, a consistently applied light source which is too intense can, in fact, cause glare, washing out the details of the surgical site and hence mitigating contrast. The relationship between contrast and glare is thus in a delicate balance, with the exact illumination threshold dependent on the specific structures and surgery at hand. In simpler terms, brighter is not always better when it comes to visualization. Therefore, targeted, modulatory lighting is recommended for parsing out the details of the surgical site and allowing contrast to inform the surgeon's understanding of the anatomy. In this way, a base level of lighting which is moderately intense, with the option to apply enhanced high-intensity, buildable light for specific subtasks, is preferential for balanced visualization throughout the surgery.

In line with the effects on visualization, high-intensity, conventional OR lighting can also adversely affect surgeons' health and performance over time. Photoreceptors in the eye are highly sensitive to stimuli, and may be affected depending on the duration, intensity, wavelength, and intermittence of light. Surgeons are particularly vulnerable to such effects, as surgeons work under illumination conditions that are high-intensity and long duration, over multiple years. In the short term, this condition manifests itself as eye fatigue, or the general symptoms of mild pain, headache, and sensitivity around the eyes. Indeed, studies have suggested that extended exposure to high-intensity light in the OR may contribute to eye fatigue specifically in surgeons. 3 In the long-term, sustained exposure to nonmodulated illumination sources may result in permanent photochemical damage, as the eye loses its ability to protect the retina. The adverse effects of hyperintense light sources on surgeons are beginning to be recognized in the literature. For example, in one meta-analysis of over 5,000 surgeons, Stucky et al found that over 25% of surgeons reported eye strain as an occupational health hazard. 4 OR light source was one of multiple OR ergonomic factors considered. Future studies should aim to capture the effects of light-derived eye fatigue and strain among surgeons, to measure the long-term impact. Therefore, moderate, buildable light with the option for directed, enhanced illumination is cited as optimal for surgeons to promote visualization, as well as reduce eye fatigue and strain.

Further analysis corroborates that central issues with overhead SLS derive from the pendant arms, which allow the possibility of collision and/or drift, and also contribute to eye fatigue due to overhead lighting. Moreover, SLS represent a potential source of contamination by way of the surgical light handles, despite coverage with sterile light handles or sleeves. Schweitzer et al revealed that in hip replacement surgery procedures in their hospital, 50% of randomly selected sterile light handles contained a significant amount of bacterial culture. 5 Given that sterile surgical light handles are often manipulated during the procedure to expose the patient to more or less light, there exists the potential to transfer bacteria between the light handle, the surgeon or adjuster's gloves, and the wound site, particularly if luminaire adjustments occur often throughout the course of a procedure.

Practicing surgeons concur with the above issues, and further add concerns on the clinical experience of alternative light sources, such as headlights and lighted retractors. Qualitative survey analysis of 12 breast surgeons concluded that 92% of surgeon respondents did not prefer to utilize headlights during surgery, citing insufficient light for deep cavities, presence of shadows and glare, head and neck strain, continuous adjustment, and potential source of contamination as reasons for nonpreference. 6 Other complaints traditionally have been the unwieldiness of cables related to lighted retractor systems on the surgical field, as well as the tethering of a cabled headlight to the surgeon, limiting mobility. Surgeon respondents' main contentions with fiber optic lighted retractors centered on heat concerns and the perception that lighted retractors provide less than optimal lighting.

In the current illumination landscape, there exists an urgent need for a light source that is nimble, sterile, functionally simple, and visually superior. In the words of Knulst et al:

“A sound surgical lighting solution will provide always good illumination at a wide range of locations simultaneously, thus minimizing the need for and effect of luminaire repositioning. As small-entrance deep wounds were reported to be difficult to illuminate, the development of tailored lighting solutions might be advisable for these cases . . . the surgeon should be able to perform this task with minimal effort and by paying minimal attention to this secondary task.” 2

### Takeaways

- The main illumination methods used in surgery are OR lighting, lighted retractors, headlights, and operating microscopes.
- Each method carries advantages and disadvantages driven by function and ease of use.
- OR lights are frequently adjusted, notably every 7.5 minutes, and 50% of previously sterile light handles have been shown to foster bacterial growth.
- Light which is too bright can introduce glare, which thus impedes the ability to appropriately visualize contrast.
- Similarly, too-bright light can result in strain, fatigue, and permanent photochemical damage to surgeon's eyes.
- Practicing surgeons contend issues with current options and express interest in more innovative, effective light sources.

## Safety

### Burns and Fires

The risk for burns due to light sources during surgery is well-documented in the literature, inclusive of fiber optic light cables, headlights, overhead OR lights, and operating microscopes. Fiber optic light cables, such as those attached to headlights or lighted retractor systems, are largely considered in the literature to be a major burn risk due to the propensity to record temperatures as high as 437°F. 7 Case reports of patient burns due to contact with fiber optic cables are described. Headlights themselves and even overhead OR lighting are also recorded as causes of patient burns. Operative microscopes pose a significant thermal injury risk to patients, as the working distance between light source and surgical site is relatively small, thus increasing the energy absorbed by the patient and hence the skin's vulnerability to burn. Surgical fires are an additional inherent risk of surgical illumination, specifically with regards to fiber optic light cables. Strategies to avoid burns and fires, as well as the advantages and disadvantages of such strategies, are also discussed.

“While the healthcare community has made great strides in preventing surgical fires, we must not be complacent.” 8

-Scott Lucas, PhD, PE, Director of Accident and Forensic Investigation at ECRI Institute

Multiple clinical case studies report on burns caused by fiber optic cables, headlights, and/or overhead OR lights. Fiber optic cables and cords are often attached to lighted retractors or headlights, and thus used in illumination among a great number of surgical specialties, fields, and procedures. Fiber optic cables are subject to achieving dangerously high temperatures, recording as high as 437°F within 10 minutes. 7 Sandhu conducted a quantitative study in which light cables were measured for temperature as well as propensity to cause skin burns. 9 It was found that in an orthopaedic surgical simulation, light cable ends were recorded at a temperature of 213.8°F and subsequently could cause skin burns within a time horizon of seconds. This result was supported in further studies. 10 To study the effects of light cable ends in a simulated OR, Smith and Roy employed a study in which a 300-W light source was connected to a conventional fiber optic cable and placed in various positions, in contact with standard surgical instruments and items. 11 It was calculated that the fiber optic cable in contact with a surgical drape resulted in a hole in the drape within 15 seconds.

To include viable patient-centered outcomes in this exploration, Spradling conducted a comparable study utilizing cadavers as a conduit for examining skin damage due to cables. 12 In this study, the temperature for cables was recorded at 382.1°F, surpassing that of the previous study at the same unit of power. Contact with the fiber optic cable resulted in skin damage to the cadaver, despite little visible change to the drape covering the cadaver. No live or simulated patients were included in the previously cited studies, therefore the limitations of these results are that the probability of thermal damage was observed within the framework of the cable's ability to penetrate protection of the simulated patient's skin, as opposed to measuring the impact to the skin itself. Future studies may consider the inclusion of advanced skin models as a vehicle for quantifying the specific time-dependent impact of incendiary cable ends to the patient. The prior studies do, however, signify judgment on the hazards of fiber optic light cables connected to a light source, by indicating that cable ends can convey a serious burn threat in the OR, including cutaneous burns. 13 Fiber optics have been identified in a recent medical device safety report as one of the top 10 technology safety hazards. 14

Of the light sources, xenon light is most frequently associated with instances of intraoperative burns. De Armendi et al detailed a pediatric patient who suffered a second degree burn from a fiber optic xenon headlight utilized during a neck surgery procedure. 15 In this case, the exact cause was deemed to be a lack of irrigation around the wound site, combined with an incorrect proximate distance between the lens and the site at maximum intensity. Retrospective analyses from this study were integrated into the manufacturer manual to modify future use; however, such modifications are subject to the discretion of each individual surgeon.

Burns are also cited in cases of overhead light use without effective heat shielding, ranging in severity. 16 17 18 However, current light emitting diode (LED) technology can reduce heat emission.

Operating microscopes are detailed as the cause for significant burns in patients, in large part due to the short working distances necessary in microsurgery. Schutt et al describe the propensity for operative microscopes to impart thermal damage on patients. 19 Operative microscopes were measured for irradiance at varying intensities, spot sizes, and working distances. It was ascertained that microscopes have the potential to transfer large amounts of energy to the patient, measuring as high as 736.26 J absorbed by 1 cm ^2^ of skin at a working distance of 20 cm over 200 minutes. These conclusions are corroborated by the clinical literature. Choudhry et al reported a single case of a pediatric patient whose brachial plexus palsy correction surgery resulted in a first degree burn from an operating microscope. 20 Similarly, Al-Qattan and Clarke reported a case of a patient who experienced a burn following brachial plexus reconstruction. 21 In response to published Food and Drug Administration (FDA) reports that listed over 80 cases of tissue damage related to operating microscope burns, Latuska et al conducted a retrospective case review in two tertiary academic centers. 22 This study unveiled four cases of microscope-related soft tissue burns during otologic surgery. Boldrey et al supported these findings with the addition of 12 patients that suffered macular and paramacular burns as a result of light overexposure in cataract surgery. 23

Preventive methods discussed with respect to operating microscopes encourage the utilization of the lowest light intensity. 20 Yet, lowering the light intensity has the effect of reduced visualization for the surgeon, which could contribute negatively to surgical performance. Others recommend the adjustment of the aperture to align with the operative field. 22 However, illumination required in microsurgery presents a unique issue in that the tissues being operated on are typically less than or equal to 3 mm in diameter. For microsurgeries such as those detailed above, it is often infeasible to repeatedly adjust the microscope when operating on relatively small geographic areas. Lastly, the application of wet surgical sponges to the wound site can reduce the risk of burn. 22

In response to the increased reporting of light-related burns, institutions such as the FDA and the Japan Council for Quality Health Care have established registries to collate episodes of patient burns as related to light sources. 24 However, these registries are voluntary and thus often under-report the total prevalence of burns. Organizations such as the ECRI Institute have also produced guidelines for the management of light sources in surgery to avoid burns, to little measured effect. 25

There are multiple models as to how specific light sources may cause burns. The most common source of burn results from maximum intensity and overexposure, which can be controlled by selecting for lower intensity lights. 15 22 Other factors can increase the likelihood of burn. The patient's interaction with certain anesthetic agents is found to reduce the skin's ability to dissipate heat across the epidermis. 20 Choice of anesthesia can be controlled to some extent, but standard anesthetic agents may not be feasibly removed from use. Other factors include the improper draping of the patient, particularly around the wound site which receives the greatest amount of light. Inadequate draping can lend itself to increasing the surface area that is vulnerable to becoming overheated, thus increasing the burn risk. 15 A subsequent crucial factor for assessing burn risk relates to the aperture size and distance from the illumination site, which varies considerably on a light source basis. In general, it is cited that a greater distance between the light source and wound site diminishes burn risk; however, this also reduces the surgeon's visibility and is potentially detrimental to clinical performance. 9 19 20 In effect, the modifications required to mitigate the risk of burns associated with conventional light sources are viable, but not often easily integrated into standardized surgical procedures and may be slow to adopt from the practitioner perspective.

A French systematic review concluded that surgical fires caused by energy sources comprised 11% of adverse events related to health care over 6 years, indicating a significant driver for fire risk assessments in the OR. 26 In a cross-sectional study among members of the American Academy of Otolaryngology—Head and Neck Surgery, the most frequent sources of ignition for reported fires included electrosurgical units, lasers, and/or cable cords. 27 Furthermore, fiber optic light cables are broadly implicated in the “fire triangle” of the OR, serving as the heat source element. 28

Burns and fires, which are detrimental to patient safety as well as safety of all OR physicians and staff, are thus presented as a significant environmental hazard, particularly with regard to fiber optic light cables and light sources derived from xenon bulbs.

### Takeaways

- Patient burns are recorded due to contact with fiber optic cables and/or light overexposure.
- Fiber optic cables are subject to achieving dangerously high temperatures, recording as high as 437°F within 10 minutes, and can result in a burn injury within seconds.
- The ECRI Institute reports that 550 to 600 surgical fires occur annually.

## Safety

### Surgeon Health

Surgery presents a significant occupational health hazard. Surgeons must maintain positions for an extended amount of time, deftly handling fine surgical instruments, while often carrying the added physical weight of additional gear, such as protective lead or even a surgical headlight. Specifically, regarding use of a headlight, surgeons are thus placed at risk for developing musculoskeletal disorders (MSDs), including cervical degenerative disk disease, which can thereby impair their ability to effectively perform surgeries and can shorten their career. Several reports of MSDs in surgeons are discussed, in addition to recommended interventions for reducing stressors on the surgeon. Interventions emphasize moderating the surgeon's posture and removing headlights and other additional weight where possible.

In recent years, the literature on physician health has expanded to hone in on the epidemiology of MSDs among surgeons. 29 30 31 Dianat et al performed an analysis exploring the effect of the surgical profession on prevalence of musculoskeletal symptoms. 32 It was explicated that musculoskeletal symptomatology was broadly prevalent among recorded surgeons, specifically in the neck, shoulders, and low back regions, indicating that these are areas of concern for occupational health. The noted effects were reasonably mediated by the surgeon's schedule, including number and length of surgeries per week.

Cervical disk herniation is a specific musculoskeletal health issue reported in the literature as deleterious to surgeon health. Tzeng et al reported a case series of several surgeons who presented with magnetic resonance imaging-confirmed cervical disk herniation. 33 A retrospective analysis of surgeon occupational history, combined with imaging, confirmed that wearing surgical headlights and loupes was associated with symptom onset. The surgeons in this study either sought physical therapy or underwent surgery in severe cases following the analysis. All surgeons in the institution's department were instructed to wear cervical braces during operations to mitigate risk of cervical disk disease. Similarly, Sahni et al reported a single-site study that analyzed surgeon occupational health by headlight exposure, finding that 68% of high-frequency headlight users experienced aggravated neck symptoms as compared with 38% of non- or low-frequency headlight users; additionally, 34% of high-frequency users developed confirmed clinical diagnosis of degenerative cervical disorder compared with 7% of low-frequency users. 34 In a review, Fisher et al emphasized the importance of a healthy cervical spine for optimizing surgical function. 35 He noted that the cervical spine is often manipulated during surgery to enact sustained cervical hyperflexion for needed positions, rendering it vulnerable to overuse and damage. Sustained musculoskeletal fatigue imparts significant long-term health effects, thus impacting the surgeon's ability to perform future operations. 36

The negative health effects of standing with stressors during surgery can cumulatively result in significant health issues for the surgeon. Recommendations are in development to improve the state of conditions for the surgeon. Rodigari found that surgeons who accounted intense fatigue at time of standing during surgery had 16 times the risk of developing musculoskeletal pain. 37 Rodigari recommended that the surgeon's working posture be controlled to minimize stressors such as added weight, including the removal or minimization of headlight use. In agreement, Esser et al recommended interventions to improve the ergonomics of surgery, including lighting, table height, and surgical instruments as areas of intervention. 3

### Takeaways

- Physical stressors mitigate surgical performance at least once a month.
- 66% of surveyed surgeons reported having an occupational-related MSD.
- A retrospective analysis of surgeon occupational history, combined with imaging, confirmed that wearing surgical headlights was associated with symptom onset.
- If left uncorrected, unergonomic surgical posture may result in cervical sprain and, eventually, permanent disability.
- A surgeon-directed, handheld light that does not rely on head or neck angle can be effective in illuminating the surgical site without the physical consequences of other lighting modalities.

## Safety

### Distractions

Distractions and interference occur often in the OR, spanning multiple surgical specialties. 38 39 40 41 There is emerging evidence to suggest that a direct relationship exists between surgeon exposure to distractions in the OR and a decrease in patient safety. 42 Light-related distractions are often included in broader environmental or equipment malfunction interruption categories, causing the examination of its effect on surgical performance to be opaque. However, in recent years, the literature has expanded to include light adjustments as a specific subcategory of OR distractions and interruptions. Building off foundational texts, it is shown that light adjustments comprise a significant portion of interruptions during surgery and may have quantifiable outcomes on surgical performance as well as patient safety at large. Light sources thus represent an area of opportunity to significantly minimize equipment-related distractions, thereby enhancing patient safety and quality of care outcomes.

Distractions in the workplace can have long-lasting effects. Research cites that after an interruption, it can take up to 23 minutes to recover in terms of concentration and productivity. 43 The surgical theater is no different. In the OR, phone and/or pager calls are cited to be the most frequent interruptions, 44 45 46 47 48 49 50 51 and subsequently are the greatest studied. Previous studies have exhibited the prevalence of phone and pager calls as vehicles for distraction in the OR. 46 In light of this phenomenon, several studies have sought to examine the effect of phone-based distractions on specific clinical performance. In a simulation study, Yang compared simulated laparoscopic performance between surgeons who were exposed to scheduled phone call distractions and a control cohort consisting of surgeons who did not have distractions during the allotted time frame. 51 It was calculated that exposure to distractions was associated with worse surgical performance, as measured by time to completion and accuracy of the task. Furthermore, observed surgical and cognitive errors increased in the distraction cohort, emphasizing that exposure to distractions has a cumulatively negative effect on surgical performance over time.

The literature on lighting-specific distractions in the OR is limited but increasing. In 2010, Knulst et al addressed the need for observing and quantifying luminaire actions, so far lacking in the surgical literature. 2 In this study, the authors describe a method for observing OR staff during procedures, including annotations by live observers as well as video recording. The function, duration, and features of the luminaire action were noted as per a standardized rubric. The luminaire actions were also mapped on a 3D rendering to ascertain if an adjustment was made on the shortest route by distance. The authors subsequently released a questionnaire to all participants, requesting information on the respondent's perception of SLS as a whole. In this study, 56% of all lighting adjustments did not take the shortest route to completion, thus increasing the time of adjustment. It was calculated that on average, one light adjustment was noted to occur every 7.5 minutes. In 97% of the cases, the performing surgeon paused his/her task to complete the lighting adjustment. Moreover, the majority of lighting adjustments occurred during the time at which surgery was performed at the wound site, suggesting that such adjustments had the potential to viably affect specific time points in the surgery. This study demonstrated that lighting adjustments are frequent during surgery, and significantly interrupt the surgeon's actions during the operation. The findings were validated by surgeon questionnaires, which affirmed that the lighting of deep wounds and shadows is a significant issue during surgery. It has been suggested that a biometric study could yield further validation of the impact of distractions.

### Takeaways

- Emerging evidence suggests that a direct relationship exists between surgeon exposure to distractions in the OR and a decrease in patient safety.
- 63% of resident trainees made one unsafe clinical decision when distracted by cell phone and/or pager interruptions.
- After one interruption, it takes an individual on average 23 minutes to fully regain his/her concentration to the task at hand.
- One light adjustment occurs every 7.5 minutes in the OR and, therefore, is a potential source of distraction.
- In 97% of the cases, the surgeon paused his/her task to complete a lighting adjustment.
- Equipment and OR environment distractions were found to be the greatest interference factors affecting OR team function.

## Disposables versus Reusables

Reusable instruments, including lighted retractors and light cords, require a high degree of decontamination and sterilization after use, to ensure that biological materials from one patient do not come into contact with the next. Manufacturers often provide manuals to inform sterilization processes for specific instruments, and hospitals may have individual protocols in tandem. However, studies show that the decontamination processes are not 100% effective, resulting in a significant proportion of instruments that are culture-positive moving into the next procedure. Incomplete decontamination has specific effects on patient's outcomes. A retrospective study showed that the cause for a surgical site infection (SSI) epidemic in one facility was bacteria retained by reusable surgical instruments. Further case studies of infection related to reusable surgical instruments are also described. Disposable instruments offer a solution to this issue, by ensuring complete and total sterilization. Comparative studies have corroborated this statement, showing that surgeries performed with disposable instruments result in a significantly decreased infection rate. Furthermore, specific studies have combined a disposable piece, such as a sheath, with a reusable instrument and concluded that optimal sterilization was achieved. Disposable instruments, and specifically disposable additions to instruments, are thus shown to serve as an important tool for achieving quality care and patient safety outcomes.

Cleanliness is at the core of surgical performance. The decontamination and sterilization processes dictated for surgical instruments, including surgical lighting such as lighted retractors, are established, yet not consistently adhered to. 52 53 54 Southworth conducted a comprehensive literature review, returning 21 cases of incomplete decontamination. 54 Even when followed, the sterilization of reusable devices can be ineffective. 52 Kumar et al reported on steam, plasma, and ethyl oxidization routes of sterilization for reusable instruments. 53 It was evaluated that an average of 5% of steam sterilized instruments and 3% of plasma sterilization instruments failed the quality control indicators for effective sterilization.

Infection caused by the transfer of bacteria can lead to less successful postoperative patient outcomes. *Infection Control Today* highlighted a comparative study that examined the safety and efficacy of reusables and disposables. 55 Following sterilization, 29.5% of samples from reusables devices tested bacteriologically positive, of which the majority were pathogens. The FDA has also established MAUDE, or Manufacturer and User Facility Device Experience, a central database that stores reports of adverse events due to medical devices, including the improper decontamination of reusable devices. 56 Tosh et al presented a case series of arthroscopic procedure patients who were exposed to *Pseudomonas aeruginosa* and subsequently acquired SSIs. 57 In a retrospective analysis, it was revealed that the SSIs were likely related to instrument reprocessing, as multiple surgical instruments were positive for *P. aeruginosa* . It was hypothesized that minor amounts of trace tissue retained in specific instruments may have allowed an environment for the bacteria to outlast repeated sterilization. Vijayaraghavan discuss an episode in their hospital in which a *Mycobacterium chelonae* outbreak was recorded in 35 patients who underwent laparoscopy over a period of 6 weeks. 58 In a study of portable medical equipment in the emergency department setting, Obasi cultured the equipment after standard manual decontamination, to determine the efficacy of the decontamination process. 59 In this study, 25% of the tested objects yielded culture-positive results, including the presence of clinically significant microorganisms.

Disposables offer a solution to issues of contamination and sterility related to traditional reusable instruments. It is shown that the implementation of disposable instruments results in decreased infection rates postoperatively. Studies on lumbar fusion, total knee arthroplasties have demonstrated significant reductions in infection rates when switching to disposable instruments. 60 61

Evidence for the use of specifically disposable additions to reusable instruments is promising. Recognizing the intensive time and labor associated with processing reusable endoscopic instruments, Alvarado et al introduced a transparent protective sheath that did not markedly impair visualization for use on nasopharyngoscopes. 62 The instruments were tested for presence of bacteria via culture prior to the procedure, immediately following the procedure with use of a sheath, and after an extensive sterilization process which included an enzymatic rinse and ethanol disinfection. The number of instruments with culture-confirmed bacteria decreased significantly following addition of the sheath and was reduced to zero after the additional decontamination stage. The study provided support for the use of disposable sheaths that can be applied to instruments as a vehicle for contamination reduction initiatives. Indeed, such evidence has produced policy changes abroad. In the United Kingdom, a significant increase in variant Creutzfeldt-Jakob disease led the Department of Health to institute a mandatory transition to universal disposable instruments in all surgical offices performing adenotonsillectomy, with results pending. 63

Incomplete decontamination and sterilization of reusable instruments represent a significant clinical risk for patients. Disposables are proven to significantly decrease and/or nullify pathogen growth or transfer among instruments, thus mitigating infection and thereby improving postoperative outcomes for patients.

### Takeaways

- Even when followed, sterilization of reusable devices can be ineffective.
- Following sterilization, 29.5% of reusable devices tested bacteriologically positive, including pathogens.
- In a 2-week period at one single institution, seven SSIs were caused by reusable surgical instrument contamination.
- In one institution, introduction of disposable instruments reduced the infection rate by 66%, a statistically significant difference. 61

## Cost

Cost is a significant driver of Operations Management decisions. SLS (OR lights), lighted retractor sets, and headlights each impose their own set of costs, including purchase price and maintenance fees. In terms of reusable versus disposable surgical instruments, the argument for cost is more nuanced than at first glance. The true cost of reusable instruments includes not only the acquisition price, but also expenses related to decontamination and sterilization, processing, transport, utilities, and storage. In comparison, disposable instruments typically require a single predictable expense. Several studies that conducted a ground-up cost analysis of reusable and disposable instruments found that disposable instruments were more cost-effective on a per-unit basis with all factors considered. In addition to the hidden costs of reusable instruments, time in the OR is valued. Reusable instruments require set-up and adjustment in the OR, whereas disposables require none. Time in the OR can be expensive on a cost-per-minute basis; hence, saving time also saves costs incurred. The literature suggests that a cost-effective supply chain for hospitals includes disposable items where appropriate, and the optimization of time and space.

Of note, hospitals have limited resources, and thus may only own a specific, cost-limited number of lighting systems—including OR lighting and portable systems. Therefore, a hospital's OR schedule or clinical application may be limited by the supply of light sources, rendering additional administrative challenges, including cost delays.

## Current Illumination Methods

Overhead OR lights exist in every standard OR environment as a necessary minimum for surgical illumination, and thus there is a baseline cost associated with these devices. The SLS, commonly referred to as an OR light, is composed of two parts, the light system itself and the surgical light handle utilized to adjust the direction and distance of the light. The light configuration may be a single light or multiple light configuration, all of which is attached to a suspension arm or arms tethered to the ceiling, wall, or an external mobile shelf unit. Two types of lamp categories exist in OR lights: conventional, or incandescent, and LED lamps. Incandescent lamps refer to a quartz, xenon, or tungsten bulb that is filled with halogen, whereas LEDs are driven by electric currents. Current price estimates list the purchase price for the light system at $2,000 to $37,000 for incandescent lights, and $12,000 to $89,000 for LED lights. a The light system is typically sold separately from the light handle and the sterile light handle covers. The light handles usually are priced at approximately $100 to $150 for one aluminum handle, compared with $30 for one plastic handle. The sterile light handle covers are sold in large quantities, typically 120 or more per package, and are priced at approximately $200 for the total package, or about $1.50 to $3.00 per cover.

OR lights are typically high functioning for 3 to 10 years, depending on the warranty, after which maintenance and repairs will require significant investment. Maintenance and repairs of overhead lighting systems are usually serviced by a third-party vendor. Surgical lighting technician rates sit at approximately $400/h for the first technician hour, and $200/h for additional time, noninclusive of parts. Alternatively, hospitals can arrange long-term contracts with technician vendors, which cost approximately $5,000 to $6,000 per year and include semiannual and as needed technician visits. In any case, replacement parts are sold separately, purchased either from the technician vendor or directly from the manufacturer. Light system parts range in expense. Minute hardware parts are in the cents and dollars range, but specific parts, such as master controls, ceiling plate parts, and power supplies and electrical parts, can be several hundred to thousands of dollars. In terms of the lamps themselves, replacement halogen bulbs range from $10 to $100 per bulb depending on the specific features and are less expensive in bulk. Replacement bulbs are typically not necessary for LEDs, given the longevity of the source. Of note, halogen bulbs usually last 1,500 hours but can last up to 4,000 hours, whereas LED bulbs usually last for 40,000 to 50,000 hours. Therefore, the hospital's specific surgical load will dictate how often the light system is in need of replacement bulbs. Again, however, the overhead OR lights are typically a basic standard in OR suites, and their expense, in one range or another, cannot be avoided.

Lighted retractor sets are a relatively recent addition to the field. Lighted retractors broadly come in two forms, a standardized retractor that has a port for fiber optic cable connection to a halogen or LED light source, and a cordless retractor with an integrated, battery-powered LED light source. The associated costs for lighted retractor sets are as follows: the standard reusable retractor ranges from $200 to $1,500 for a single retractor, fiber optic cables range from $300 to $1,300 for a single cable depending upon the length and port size, a sterilization tray is usually around $1,000, and external light sources can range from $1,000 to $5,000 for LED, and $5,000 to $20,000 for halogen. In total, a sophisticated system can cost more than $13,000 all included. Furthermore, specific add-on pieces are available on a per case basis to enhance illumination. These single use pieces may cost between $250 and $500. Lighted retractors therefore can represent a significant expense on a per system basis, especially with the understanding that if a single retractor system is being utilized in a surgery, for example, it cannot be used elsewhere until it is fully decontaminated and sterilized again.

Headlights can be advantageous, potentially reducing the need for multiple overhead light adjustments. Headlights also minimize shadows, thus optimizing surgeon visibility. However, headlights, as noted in the Surgeon Health (IIb) section above, can also be disadvantageous. Surgical headlights are produced in a variety of light sources and configurations. Light sources include halogen and LED sources. Configurations range from the traditional, e.g., the headlight apparatus connected by fiber optic cable to a light source, to more modern iterations. Headlights are also manufactured as cordless, battery-operated, or rechargeable devices in an initiative to address portability concerns. In terms of list price, the majority of headlights range from approximately $1,000 to $10,000 per system depending upon the product specifications, the sophistication of the system, and whether or not accessories are included. In terms of maintenance, traditional standalone light sources range from $500 to $8,000 per system, and batteries required for newer headlight systems range from $100 to $200 per unit.

Novel iterations of handheld lighting technologies provide a look into the future of surgical illumination. Examples include a single cable system, which removes the intermediary cable and directly connects a LED light source with an extended length, disposable light source, and innovations such as sterile light strips that are adhesively attached to the retractor and cabled to an adjacent light source. Sterilized, disposable light strip products are typically sold in multiple-unit packages at a unit price of approximately $100 per strip, not inclusive of the cable and light source itself.

A multitude of facilities may only possess one to two ancillary lighting systems at one time, given the significant cost per unit, as stated prior. Moreover, the hospital may also be limited in the number of portable light sources that are readily available. This results in a limited supply of light sources for OR teams, given the length of surgeries. Increased demand and limited resources may thus require hospitals to secure loaner sets from another health care facility or third-party vendor, if necessary. However, the loaner set process translates into its own set of challenges and expenses. Administrators are needed to coordinate the loaner set process, which requires time as well as additional compensated staff responsibilities. Furthermore, personnel must be routed to complete the inventory, sterilization, and quality control processes for the loaner set, incurring further costs. Solutions are needed to mitigate administrative delays and ensure that surgeries can be performed with sufficient illumination using ergonomically efficient solutions.

## Sterilization and Decontamination

The cost of sterilization of an instrument tray includes the unit material cost as well as labor. For reference, LaBove et al implemented a cost analysis of a plastic and reconstructive office-based surgical suite, accounting for surgical supply, labor, and administrative costs. 64 Subsequently, the data suggested that the estimated cost of sterilization in this site, including sterilization supplies and labor, was an average of $94.28 per case. Specific procedures of abdominoplasty, facelift, breast augmentation, and liposuction were included in the average analysis. This result was validated by the conclusions of Isaacson et al, who calculated the cost for reprocessing at $96.13 for reusable flexible ureteroscopes. 65 Further estimates have calculated the comprehensive sterilization cost equates to approximately $51 to $77 per tray. 66 67 Sterilization, including sterilization of light sources such as lighted retractors, can therefore incur significant financial costs in terms of a hospital global budget, particularly in cases of incomplete sterilization. In sum, the literature suggests that the cost for reprocessing a single surgical instrument tray, inclusive of labor, can range from $51 to $96, which is not an insignificant cost when factored into the operations workflow management of a high-volume surgical center.

Given the data on sterilization, certain categories of disposable instruments are shown in the literature to be more cost-effective for hospitals. Mager et al completed a prospective clinical outcomes and cost study of procedures in a tertiary referral center, comparing reusable to single-use flexible ureteroscopes. 68 The global cost analysis included the initial purchase, repair, and reprocessing expenses for the reusable devices, compared with the acquisition price of disposable instruments. It was concluded that the cost per procedure for reusable ureteroscopes ranged from $1,212 to $1,743, depending upon the procedure. In contrast, in this institution, the minimum price of a disposable ureteroscope was $1,300. The stated results reflected a viable cost savings for the election of single-use instruments. In a parallel study, Yang et al explored cost and performance for biopsy forceps in gastrointestinal endoscopies, including purchase price as well as expenses for reprocessing. 69 It was calculated that the total cost per use for a single reusable forceps was $58.06, while disposable forceps were each acquired at $38.00. Based on the cost analysis, and comparable clinical outcomes, this institution elected to develop a strategy-driven approach, wherein disposable forceps were preferred at a certain threshold of procedure demand. In a study of fiberoptic flexible scopes for difficult tracheal intubation, Aïssou calculated that the differential costs between reusable and disposable scopes were minimal when acquisition, sterilization, and maintenance expenses were included, €206 as compared with €200, respectively. 70 Based on these results, the authors indicated a preference for proceeding with single-use devices. Cost equivalency and additional researched benefits of disposable devices were cited as the basis for the decision.

## Additional Considerations

*Separate from the sterilization process, time in the OR is valued on a cost-per-minute basis.* Yu et al conducted a time-driven activity-based costing of pediatric appendectomies, including consumable and labor costs. 71 The cost per minute in the OR was found to be $25.55 for this procedure. Similarly, Childers and Maggard-Gibbons performed a review of hospitals in California, completing analysis that the mean cost per minute of OR time was $37.45 for inpatient and $36.12 for ambulatory procedures. 72 In a study of New York health systems, Girotto et al concluded that the cost per minute in the OR was between $60.00 and $100.00. 73 As evidenced above, any additional time in the OR is cost wasted, and elements such as set-up time and time required to adjust, for example, fiber optic cable attachments or overhead light handles, merely increases that cost, contributing to delays and poor efficiency. A further expense to consider is that of storage allocation in the hospital or surgical suite. Conventionally, reusable instruments are sterilized and stored on-site when not in use.

### Takeaways

- Acquisition cost of ancillary surgical lighting can be expensive, with a purchase price of up to $10,000 to $13,000 for advanced headlights and lighted retractor systems.
- Maintenance and repair costs are often overlooked, and average $5,500 per year for technician time, and can cost up to $1,000 per year in replacement parts depending on the specific part.
- Sterilization and processing costs for reusable instruments are additional real costs and are estimated at $50 to $100 per tray.
- The average cost per minute in the OR can be up to $100 per minute, not including the surgeon's cost, so efficiency and time savings can result in an increase in facility profitability.
- There is further value to optimizing shelf and storage space, by minimizing additional costs.

## Conclusion

The evolution of surgical illumination continues to be addressed through research and practice. However, the literature lends itself to providing a framework for assessing the needs of surgeons with respect to surgical illumination. Three components of surgical lights are essential: a light source should (1) center on the surgeon's immediate field, (2) illuminate with high-intensity light, and (3) viably penetrate into surgical cavities or under flaps. Each of the current OR lighting methods meets at least one of these criteria, but none meets all, thus leaving room for a novel product to enter the space.

Researchers and surgeons alike contend several issues with conventional light sources. Burns and fires represent a significant risk of current lighting systems. Surgical lighting, most frequently fiber optic cables from lighted retractors, are directly responsible for severe burn damage to patients, as recorded by the U.S. FDA. Patients have suffered second and third degree burns as a result of current lighting options. Light sources are also implicated in a great proportion of surgical fires, which serves as an environmental hazard for patients, surgeons, and all OR and hospital staff.

In line with safety and workflow concerns, current lighting systems take their toll on surgeons with respect to physical and cognitive health. Headlights specifically impart ergonomic issues, in large part due to the weight and need for movement-driven adjustment. Studies have reported that frequent headlight use is an occupational health hazard with specific negative health outcomes and may even be linked to the shortening of a surgeon's career. More broadly, current lighting systems are associated with multiple levels of adjustment, from moving surgical light handles to the alteration of cables for lighted retractors. Aside from data which reveal that even previously sterile OR light handles harbor bacteria, and the extrapolation that repeated manipulation can result in transfer to surgical gloves, such distractions have a marked effect on surgeon performance, with experts citing that a 1-minute distraction may result in a 23-minute delay in cognitive processing and focus. Distractions could lead to negative outcomes with respect to patient safety and quality of care. Future biometric studies may explore in-depth impact of specific distractions on surgeon's performance and OR ergonomics, providing a research tool to support use of future OR technologies.

On another note, time also has measurable outcomes on global cost to the hospital. Cost-per-minute in the OR varies from hospital to hospital as well as regionally but can be as high as $100. Given that each adjustment of a lighting system can take minutes, multiplied by the total number of adjustments per surgery, it logically follows that cost associated with light-related distractions may represent an unnecessary expense to the hospital.

With respect to future surgical illumination sources, it is debated whether reusable or disposable options are most advantageous. This can be considered from the sterility and cost perspectives. With respect to decontamination and sterilization, disposable instruments can be more effective than reusable instruments. Multiple cases of measured nonsterilization are reported with reusable instruments, with marked effects including SSIs. In addition, in many cases, when all hidden costs are factored in, it is found that disposable devices are, in fact, often less expensive on a per-unit basis, strengthening the support for single-use instruments, including lighting devices.

## Footnotes

a Prices presented as list price and may differ from negotiated contract rates.
